# An Approach to Building Equitable Global Health Research Collaborations

**DOI:** 10.5334/aogh.3039

**Published:** 2020-10-01

**Authors:** Megan Murray, Joel Mubiligi

**Affiliations:** 1 Department of Global Health and Social Medicine, Harvard Medical School, Boston, MA, US; 2 Partners In Health/Inshuti Mu Buzima, Kigali, RW

In an ideal world, it would be simple and mutually rewarding for researchers in high-income countries (HICs) to collaborate with researchers in low-income countries (LICs) to conduct high-quality research to improve health outcomes. Surely, the abundance of intellectual, technical, and financial riches in HIC universities could be harnessed to the experience of “local” clinicians, using “local” here to reference those working in the context studied, to bring about productive and equitable partnerships that further the missions of both groups. In reality, however, it is inevitable that the needs of researchers in HICs and LICs sometimes diverge. Even when both groups are dedicated to improving the health of the most vulnerable, they are each embedded in systems that operate under different constraints and through different incentive structures. Here we outline some of these issues and offer our long-term approach as a jointly beneficial strategy to redress these challenges.

For “local” implementers, the decision to conduct research is weighed against difficult trade-offs. Given limited personnel, facilities, and materials, research comes at short-term costs to patient care, and it is often difficult to predict the long-term value. Scarcity is not only a factor in prioritizing healthcare activities; relatively few LIC healthcare professionals have the opportunity for research training, and access to medical literature is limited by language, technology, and the cost of journal subscriptions. These barriers make it challenging for even the best-trained LIC researcher to effectively compete for funding.

These issues mean that many partnerships are inherently unequal. Successful HIC researchers are highly-trained, have access to medical literature, and understand how to obtain grant funding. Relatively few face the trade-offs and time constraints that LIC researchers encounter. This can result in HIC researchers setting the agenda of “collaborative” research, often choosing research questions that are fundable but less relevant to the local setting.

HIC researchers face very different constraints. At most HIC universities, faculty need to bring in grant funds to finance their research. The majority of these applications are rejected. For example, in 2014 the NIAID set the payline for an R01 (a five-year investigator-initiated award) at only 9%. Many grants are reviewed by HIC-based experts – usually without first-hand experience in the LIC in which the project will take place – who assess applications on the basis of the “excellence” of an investigator’s previous work, the proposed methodological or technical “innovations,” and the “significance” of the project as it is perceived in a HIC. Much recent commentary has focused on the limitations of the ideal of excellence in science, how it “reinforces disciplinary boundaries and focuses scientists’ attention inwards rather than on the problems of the outside world [1,2,3].” But even as the compelling case “against excellence” is mounted, researchers seeking funding often find themselves locked into a system that rewards “cutting-edge” science, at the expense of research that may have a more meaningful impact on the most vulnerable populations.

The path to promotion in academic global health and medicine is also fraught with obstacles. Career advancement for academic researchers in HICs usually involves a series of stepwise promotions, from instructor to, finally, full professor. At some universities, it can take more than 10 years to move through these steps. For faculty involved in research, promotions are awarded on the basis of the number of first and last-authored publications, invited research presentations, impact factors of the journals in which one publishes, and funding. Global health collaborations that distribute these rewards among the individuals involved can reduce the apparent success of a HIC faculty member on an “up or out” tenure ladder [4].

On the other side, the career path for LMIC researchers is often not clear. Many clinicians are eager to gain research skills and find answers to their questions, but are without an obvious path in academic research. When they do not belong to an organization that has experience in this, many of their co-authorship agreements lack legal safeguards. These structures can lead to an asymmetry in bargaining power that erodes efforts to establish a reliance structure where reasonable expectations are fairly negotiated. This leaves the LMIC partner in a situation of vulnerability [5] where they might be forced to enter a collaboration where the other partner has greater or total influence the project’s outcome.

We have tried to overcome misalignments between the needs of LIC and HIC researchers through the approach to research and research training we have developed through a long-term partnership between Harvard Medical School (HMS) and the non-governmental organization Inshuti Mu Buzima (IMB) in Rwanda. This is a work in progress and challenges remain, but we can identify a number of ways in which some of the issues above have been ameliorated, as well as a number of areas in which we aim to improve.

First, all research has to align with the clinical mission of IMB. This is essential to avoid dissipating limited resources and effort on areas that are not relevant to the health of the community and patient care. Ideally, research should map to strategic goals that have been articulated in advance, while acknowledging the need for some flexibility to respond to the unexpected.

Secondly, we have institutionalized the Intermediate Operational Research Training Course [6] which involves a hands-on mentored research experience intended to lead to a first publication for a research team within one year. This training itself is a manifestation of our partnership. IMB leads the process of identifying research topics, prioritizing questions that link to clinical care to mitigate the trade-offs between research and implementation, and identifying the trainees best positioned to rapidly translate findings into clinical decision-making. IMB contributes individuals to the training team, particularly junior researchers who are tapped to lead this training model in the future. Historically, HMS faculty have served as the core facilitators and mentors, though the intensity of these roles decreases with every training as past trainees become trainers. The training program was first open to *any* interested IMB or Ministry of Health staff regardless of position or educational background; we have found that reducing the requirement for participation helps de-fetishize the “excellence” that can create a barrier to conducting research with potential impact. The trainee pool has expanded to include faculty from the University of Rwanda to increase our collaboration networks and future mentor pools.

Thirdly, all externally-funded research conducted at IMB is required to have a plan that will increase the capacity of their teams and IMB overall to lead future research. Most of this funded research feeds into the Intermediate Operational Research Training course, either by generating datasets for the trainings, underwriting the cost of the training, or engaging the leaders of these projects in the mentorship of new researchers. Team members from larger research projects at IMB join the training with the goal of growing the project’s research leadership for better equity in the collaborations.

Fourthly, the Intermediate Operational Research Training course has been highly productive in terms of the number of publications, with almost all teams producing a publishable paper within one year. The HMS mentor is a co-author on the publication, though often not first or last. We indicate in the publication that even when a trainer or mentor is not the first or last author, they have played a major role in the work. On the HMS-side, we have worked closely with promotions committees to stress the importance of tenure review policies that honor equitable partnerships, including mentorship “middle-author” roles, which to some degree has offset concerns from the university that junior faculty are taking career risks by ceding first and last authorship positions.

A recent example helps illustrate some of these points. In one training, Christian Mazimpaka and Eline Uwitonze first-authored a study on perioperative management and outcomes after cesarean section (c-section) in rural Rwanda [7]. The senior author had taken the course previously [8] and had become the study manager of an NIH-funded project on c-sections. Using these data, Dr. Mazimpaka and Ms. Uwitonze found that in 30% of cases, the indication for c-section was a previous c-section, since vaginal birth after c-section is not permitted in this setting. Although these surgeries could have been scheduled in advance, all of the women presented during labor and underwent an urgent procedure. As District Clinical Director, Dr. Mazimpaka acted on these results by placing ultrasounds in health centers to help estimate gestational age and better plan for c-sections. This work also professionally benefited the course director, HMS faculty Dr. Hedt-Gauthier; in addition to being a co-author on the resulting publication, she was able to cite this paper as a deliverable of her NIH-funded study and she has been included as a co-investigator on a grant led by Dr. Mazimpaka to evaluate the intervention.

The challenges facing LIC researchers and the production and dissemination of high-quality and locally relevant research are vast, and all key players must proactively engage to shake the current paradigm. For our collaboration, and similar to other capacity-strengthening programs in non-academic settings in sub-Saharan Africa [9], success is rooted in focusing on our common goal – producing high-quality information for the questions of greatest import to those in positions to affect change in programs and policy – and then designing strategies that are both effective and mutually beneficial to all involved.
